# The Substitutor

**Published:** 1892-06

**Authors:** 


### EDITORIAL DEPARTMENT.

F. E. DANIEL, M. D., EDITOR AND PUBLISHER.

THIS JOURNAL, by unanimous vote of the members, was made the Official Organ of the Austin District Medical Society.

COLLABORATORS:

*Dr. R. M. Swearingen, Austin.*
*Prof. B. E. Hadra, M. D., Galveston.*
*Prof. Geo. Cupples, M. D., San Antonio.*
*Dr. T. C. Osborn, Cleburne, Texas.*
*Dr E. J. Doering, Chicago.*
*Dr. E. J. Beall, Fort Worth.*
*Dr Odo Betz, Germany.*
*Prof. W. B. Rogers, M. D., Memphis.*
*Dr. J. W. McLaughlin, Austin.*
*Dr. T. J. Tyner, Austin.*
*Prof. J. F. Y. Paine, M. D., Galveston.*
*Dr. R. H. L. Bibb, Mexico.*
*Dr. T. J. Bennett, Austin.*
*Dr. Bat Smith, Wharton.*
*Dr. E. Meierhof, New York.*
*L. H. Luce, M. D., West Tisbury, Mass.*

### THE SUBSTITUTOR.

This, and other leading and independent journals, have had much to say against the evil of substitution by druggists, of preparations of their own make for well known compounds prescribed by physicians. In consequence of this and other evils, the JOURNAL has gone so far as to advise physicians to purchase their supplies from manufacturers and dispense their own medicines; cutting loose from the prescription druggist altogether. Perhaps we went too far, for there are some honest druggists who are above such tricks; but one cannot always discriminate.

The magnitude of the evil is not fully appreciated by physicians, because they have not reflected well upon it, perhaps. Not only a physician's reputation, and consequently his practice, may be destroyed by having an unscrupulous druggist dispense a preparation of his own manufacture, when an article of ascertained strength and tested merit has been ordered, but the life of the patient may be sacrificed. We all know that there is great variation in the strength of crude drugs, and the object of substitution being, of course, gain, the substitutor will use cheap (and therefore, worthless,) drugs in the preparation of his "just as good" counterfeit.

Much has been said upon the subject, and the profession are being aroused to the importance of specifying any particular make or brand they may want, and of seeing that it is dispensed.

About the strongest article on the subject we have seen, appears in a neat little pamphlet, published by the N. Y. Pharmaceutical Co., Bedford Springs, Mass., with the picture of a druggist with embryo horns, and a low cunning face, offering a gentleman something "just as good" of "our own make," when he has called for Hayden's Viburnum Compound. The picture is described in forcible and just terms of condemnation. From the article we are tempted to extract the following.

Some scientists have expressed the opinion that the substitutor might be a cross between the vampire and the ourang-outang, but there is no positive evidence in support of the theory, besides, it is an insult to the ourang-outang! * * The substitutor is a companion of "La Grippe," but is far more dangerous to human life; for the latter will sometimes spare his intended victims, but the former never; both are emissaries of death. * * The real intent of these substitutors is to deceive the physician, defraud the patient, filch the good reputation earned by another, substituting their own questionable preparation instead of the one desired and prescribed. Such puplicity is called, among honorable men, *swindling*. The imitator and the counterfeiter are not half as dangerous as the substitutor, for their wares are easily detected and expose themselves. The substitutor is doing a vast amount of harm; his example is pernicious in the community in which he thrives. *He is daily bringing into disrepute one of the most important professions which is as much so as that of the practice of medicine.* We trust our lives and those of our loved ones in the hands of the pharmacist, and we have confidence that he will do his part faithfully to relieve human suffering and prevent death. Not so with the dreaded and abhorred SUBSTITUTOR, for his greed urges him on in his nefarious business, to sacrifice thousands of innocent lives, that thereby he may make a few extra pennies by his cruel deceptions.

The medical press, the "molders of opinion," owe it to the medical profession, and to humanity, to denounce this most nefarious practice; to do all in their power to put a stop to it. It is one of those insidious evils, which like an octopus has fastened itself upon the body medical, and is sucking the life-blood of professional reputation. It is doing more, it is endangering, nay, destroying human life, and there is no law to prevent it. There is a law against adulteration of drugs, we believe, but not against substitution of worthless preparations for those of established merit, and these fellows seldom get their just deserts.

